# Shiga Toxin Binding to Glycolipids and Glycans

**DOI:** 10.1371/journal.pone.0030368

**Published:** 2012-02-13

**Authors:** Karen M. Gallegos, Deborah G. Conrady, Sayali S. Karve, Thusitha S. Gunasekera, Andrew B. Herr, Alison A. Weiss

**Affiliations:** Department of Molecular Genetics, Biochemistry and Microbiology, University of Cincinnati, Cincinnati, Ohio, United States of America; Columbia University, United States of America

## Abstract

**Background:**

Immunologically distinct forms of Shiga toxin (Stx1 and Stx2) display different potencies and disease outcomes, likely due to differences in host cell binding. The glycolipid globotriaosylceramide (Gb3) has been reported to be the receptor for both toxins. While there is considerable data to suggest that Gb3 can bind Stx1, binding of Stx2 to Gb3 is variable.

**Methodology:**

We used isothermal titration calorimetry (ITC) and enzyme-linked immunosorbent assay (ELISA) to examine binding of Stx1 and Stx2 to various glycans, glycosphingolipids, and glycosphingolipid mixtures in the presence or absence of membrane components, phosphatidylcholine, and cholesterol. We have also assessed the ability of glycolipids mixtures to neutralize Stx-mediated inhibition of protein synthesis in Vero kidney cells.

**Results:**

By ITC, Stx1 bound both Pk (the trisaccharide on Gb3) and P (the tetrasaccharide on globotetraosylceramide, Gb4), while Stx2 did not bind to either glycan. Binding to neutral glycolipids individually and in combination was assessed by ELISA. Stx1 bound to glycolipids Gb3 and Gb4, and Gb3 mixed with other neural glycolipids, while Stx2 only bound to Gb3 mixtures. In the presence of phosphatidylcholine and cholesterol, both Stx1 and Stx2 bound well to Gb3 or Gb4 alone or mixed with other neutral glycolipids. Pre-incubation with Gb3 in the presence of phosphatidylcholine and cholesterol neutralized Stx1, but not Stx2 toxicity to Vero cells.

**Conclusions:**

Stx1 binds primarily to the glycan, but Stx2 binding is influenced by residues in the ceramide portion of Gb3 and the lipid environment. Nanomolar affinities were obtained for both toxins to immobilized glycolipids mixtures, while the effective dose for 50% inhibition (ED_50_) of protein synthesis was about 10^−11^ M. The failure of preincubation with Gb3 to protect cells from Stx2 suggests that in addition to glycolipid expression, other cellular components contribute to toxin potency.

## Introduction

*Escherichia coli* O157:H7 is the most common serotype of Shiga toxin-producing *E. coli* isolated from patients in the United States. It is estimated to cause 110,000 cases, mostly among children and the elderly, and 3,200 hospitalizations annually in the United States, costing approximately 400 million dollars [1], [2]. This pathogen causes food-borne disease with symptom severity that varies from mild diarrhea to hemorrhagic colitis, and potentially to life-threatening Hemolytic Uremic Syndrome (HUS) [3]. Shiga toxin (Stx), the most important virulence factor of *E. coli* O157:H7, is responsible for the life-threatening complications following infection. Stx is an AB_5_ toxin consisting of a single A subunit associated with a pentamer of identical B subunits. This pentamer binds to the glycosphingolipid globotriaosylceramide (Gb3) in host cell membranes [4], [5], [6], [7] and delivers the A subunit into the cytoplasm. In the cytoplasm, the enzymatically active A subunit inhibits protein synthesis by cleaving an adenine nucleotide from 28S RNA within the 60S ribosomal subunit, preventing tRNA binding and protein synthesis [8], [9].

There are two immunologically distinct forms of Stx: Stx1 and Stx2. They share 56.8% amino acid sequence identity [10], [11]. In epidemiological studies, Stx2 is more often associated with severe disease outcome and development of HUS than Stx1 [3]. In animal models, Stx2 is 100- to 400-fold more potent than Stx1 [12], [13], [14]. Differences in host cell receptor binding between Stx1 and Stx2 appear to mediate the differences in potency in vivo and in vitro [13], [15], [16], [17], [18]. Shimizu et al. reported that a chimeric toxin with the Stx2A subunit associated with the Stx1B-pentamer was 2-fold more toxic to mice than wild type Stx1 and 50-fold less potent than wild type Stx2, suggesting that the A subunit does not significantly contribute to potency in vivo, while the B-pentamer play a more significant role [6]. These data suggest that Stx potency might be due to a differential targeting or affinity in binding to host cell receptors. When Stx1 or Stx2 is administered to mice, Stx1 stays predominantly the lungs without causing pathology while Stx2 mainly targets the kidneys [13], [19]. It has been suggested that Stx1 might bind to Gb3 variants in the lungs, preventing it from reaching more susceptible organs such as the kidneys, whereas Stx2 binds preferentially to Gb3 variants in kidney tissue.

Stx binding to the Pk trisaccharide (Galα1-4Galβ1-4Glc) present in Gb3 occurs primarily through hydrogen bonds between the hydroxyl groups on the sugars. High affinity is achieved through avidity, by engaging multiple binding sites on the toxin. The Stx1 B-pentamer has 3 Pk trisaccharide binding sites per subunit, or 15 sites total per holotoxin [20]. In contrast, the binding sites for Stx2 are less well defined, but the binding interactions have been modeled [21], [22]. Interestingly, binding studies using receptor mimics show that Stx1 binds with higher affinity to the Pk trisaccharide than Stx2 [15], [23], [24], [25], [26]. Published data demonstrate different and selective binding preferences of Stx1 and Stx2 to synthetic glycans. Stx1 shows a preference for binding native Pk while Stx2 binds better to an N-acetylated analogue of Pk (NAc-Pk) [16], [24], [27]. Native Pk trisaccharide is found on glycolipid Gb3, while NAc-Pk is found on proteins, but no glycolipids with NAc-Pk are known to exist in nature.

Native Gb3 is found on the lipid rafts (detergent-insoluble glycolipid-enriched domains) in host cell membranes. Lipids rafts are composed of (glyco)sphingolipids, glycerophospholipids, and cholesterol. Stx2 variants, such as porcine edema disease toxin (Stx2e), have been reported to bind to glycosphingolipid globotetraosylceramide (Gb4), which contains an additional residue, GalNAc, attached to the Pk of Gb3 [28], [29]. In a recent report, Gb3 was found to be present in low quantities in colonic epithelial cells in vivo; whereas Gb4 was found abundantly [30]. Low affinity binding of Stx1 to Gb4 has been reported [30], suggesting that Stx1 could bind to these glycolipids in host cells membranes. However, the true functional receptor of Stx remains unknown. It is not clear if Gb3 is the main factor mediating Stx binding to host cells, and in vitro binding affinities do not correlate with cellular or in vivo toxicity. Previous data shows that Stx affinity for Gb3 is in the nanomolar range while cellular and in vivo toxicity are in the picomolar range, suggesting other factor might also play a role of Stx toxicity in vivo and at cellular level [12], [15], [23].

Recently, it has been reported that lectin binding was enhanced in the presence of glycolipid mixtures as compared to the binding to single glycolipids [31]. Considering that glycolipids are naturally found in the cell membrane in mixtures and in combination with phospholipids and cholesterol [32], [33], Stx binding in vivo might involve more than one glycolipid, and the presence of cholesterol and phospholipids.

The objective of this study was to gain insight into the receptor preferences for Stx1 and Stx2. We examined binding of Stx1 and Stx2 to various glycans, glycolipids and glycolipid mixtures by ITC or ELISA in the presence or absence of phosphatidylcholine (PC) and cholesterol (Ch). The findings of this study have clarified the differences in binding of Stx1 and Stx2.

## Results

### Characterization of individual glycan binding sites by ITC

**Figure 1:**
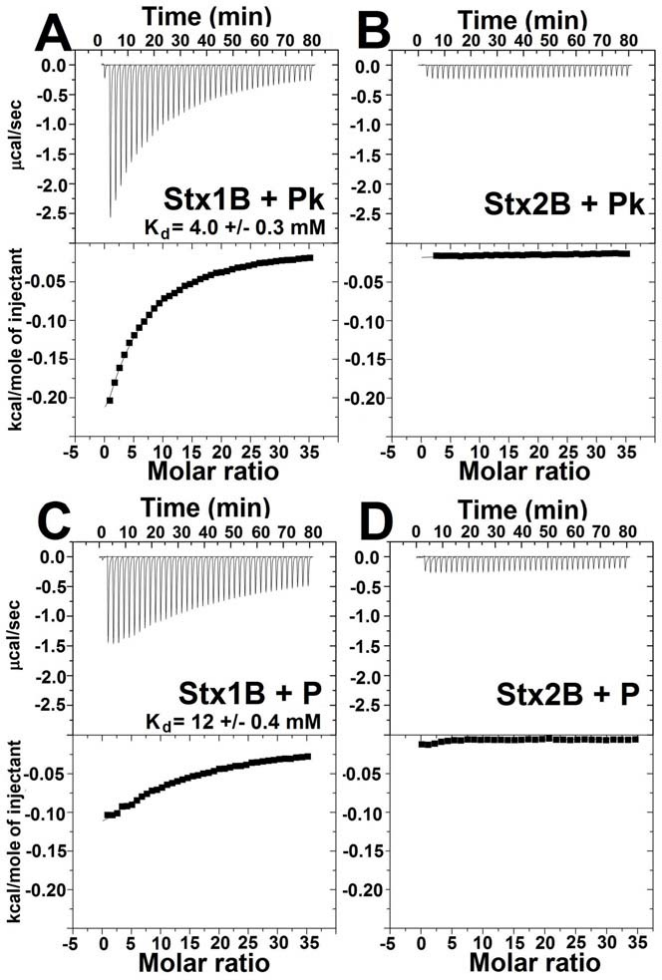
Binding of Stx1 and Stx2 to purified Pk trisaccharide and P tetrasaccharide by ITC. Glycans (50 mM) were titrated into a microcalorimeter cell containing 238–300 µM of Stx B-subunits. Stx binding interaction with Pk (A, B) and P tetrasaccharide (C, D). Both Stx1 B-subunits (A–C) and Stx2 B-subunits (B–D) raw heat signals (top) and integrated data from titrations (bottom) are shown.

While the glycolipid Gb3 is commonly reported to be the receptor for both Stx1 and Stx2, the two toxins appear to have different receptor preferences. We used ITC to examine binding of Stx1 and Stx2 to the Pk-trisaccharide expressed on Gb3. To avoid complications due to A-subunit interactions, binding studies were performed with purified B-pentamer. Stx1B bound to Gb3 with a K_d_ of about 4 mM (Figure 1A), which is in good agreement with previously published studies using ITC [34] and mass spectrometry [35]. In contrast, no binding of Stx2B to Pk was detected under the experimental conditions tested (Figure 1B).

In previous reports, Stx1 and Stx2 binding to Gb4 was observed [23], [29], [36], [37], [38] and recently Stx1 has been reported to bind to Gb4 [30]. However, nothing is known about the number or affinity of single sites for Gb4. Stx1B bound to the P tetrasaccharide expressed on Gb4 with a K_d_ of 12 mM (Figure 1C), with about 3-fold lower affinity compared to Gb3. These results demonstrate that Stx1 might recognize Gb4 as receptor. Like Pk, no binding of Stx2B to P-tetrasaccharide was observed (Figure 1D), which suggested that the K_d_ of Stx2B for both glycans is much greater than 12 mM.

### Stx binding to synthetic glycans

To identify other possible glycan receptors, Stx1 and Stx2 toxoids were assayed by ELISA for binding to 465 different glycans by the Consortium for Functional Glycomics. To avoid exposure to the high concentrations of toxin typically used in binding studies, these studies were performed with genetically inactivated toxin. The two amino acid changes (Tyr77Ser and Glu167Gln) abolish the enzymatic activity of the A-subunit, but do not affect binding mediated by the B-pentamer [39], [40].

**Figure 2:**
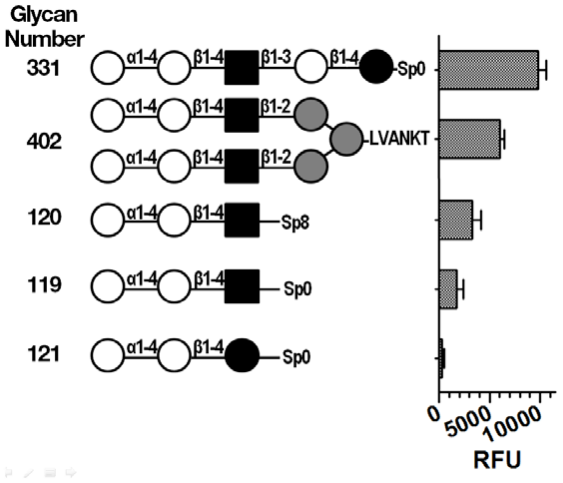
Glycan array results for Stx1. Binding of Stx1 (2.84 µM) toxoid to the Consortium for functional Glycomics Mammalian Array Version 4.1 with 465 different natural and synthetic mammalian glycans was assessed by ELISA. Displayed are the top three hits for Stx1 (glycans 331, 402, and 120). For comparison, also displayed is native Pk (glycan 121), glycan 119 which is attached using the same linker as native Pk, and glycan 120, which is attached with a different linker from 119. The symbolic representation of the compounds follows the CFG standards: galactose (Gal, white circle), glucose, (Glc, black circle), *N*-acetyl-glucosamine (GlcNAc, black square), mannose (Man, gray circle). X corresponds to β1-4GlcNAcβ1-4GlcNAcβ-LVANKT. Spacers used to couple the glycans to the array surface matrix: Sp0, -CH_2_CH_2_NH_2_; Sp8, -CH_2_CH_2_CH_2_NH_2_; LVANKT, peptide (Leucine, L; valine, V; alanine, A; asparagine, N; lysine, K; threonine, T). Relative fluorescence units (RFU) signal is the mean of four independent experiments and error bars indicate Standard Deviation (SD).

No significant binding was detected for Stx2 at 0.64 µM (data not shown). Binding to Stx1 was detected. The top three hits for Stx1 (Figure 2, glycans 331, 402, and 120) resembled Pk trisaccharide, with Galα1-4Gal as the terminal sugars; however they differed from Pk at the third sugar, which was GlcNAc instead of Glc (Figure 2). Interestingly, Stx1 did not display significant binding to glycan 121, containing the native Pk antigen. However, consistent with the above results, 7-fold more binding of Stx1 was observed to glycan 119 (the 6^th^ best hit) which only differed from glycan 121 by the presence of *N*-acetylation at the third residue, suggesting GlcNAc may be the preferred residue. However, in nature, this glycan (Galα1-4Galβ1-4GlcNAc) occurs in mammalian glycosylated proteins, but not on glycolipids.

Additionally, the linker used to attach the glycans to the array surface matrix can influence toxin binding [24], [41], [42]. Glycan 120 and glycan 119 share the identical glycan trisaccharide, but are attached with different linkers. A change from the Sp0 linker (-CH_2_CH_2_NH_2_) to the Sp8 linker (-CH_2_CH_2_CH_2_NH_2_) increased Stx1 binding by 2-fold.

### Stx binding to native Gb3 glycolipid

**Figure 3:**
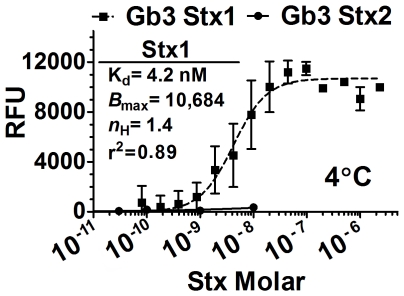
Stx binding to pure Gb3. Stx1 (black squares, ▪) and Stx2 (black circles, •) toxoid binding affinity to Gb3 alone was assessed by ELISA at 4°C. Stx1 binding as fitted to a one-site specific binding model with Hill coefficients. Symbols represent experimental data, while lines represent the fitted model for that data analyzed with Prism5 (GraphPad software, La Jolla, CA). Values for Stx2 were not determined due to poor binding. The RFU signal is the mean of three independent experiments and error bars indicate SD.

The failure of the glycan array to reveal binding of Stx1 to native Pk, and the inability to detect any ligands for Stx2 led us to examine binding to native glycolipids. In initial experiments, binding at several concentrations of Stx was assessed using pure Gb3 immobilized on hydrophobic microtiter ELISA plates with incubations at 4°C. The apparent dissociation constant (K_d_) of Stx1 binding to Gb3 was determined to be 4.2 nM (Figure 3) which is 10-fold lower than the 46 nM value reported with radio-labeled Stx1 and 48-fold lower than the 222 nM value reported with Surface Plasmon Resonance (SPR) [15], [23]. There are several explanations for the different apparent K_d_ values obtained in different studies. ELISA has been shown to be more sensitive than SPR [16], [43], possibly because the longer incubation periods in the static ELISA allows the toxin to achieve optimal interacting conformation compared to the dynamic flow conditions of SPR [42]. Additionally, we incubated the plates at 4°C, while the SPR studies were done at room temperature.

In contrast a K_d_ for Stx2 binding to Gb3 was not determined due to the high concentration of toxin (above 1 µM) needed to reach saturated binding under these conditions (Figure 3). Previous studies reported low affinity binding of Stx2 to Gb3 using radio-labeled Stx2 (K_d_ = 370 nM) and SPR (K_d_ = 1040 nM) [15], [23].

### Stx binding to glycolipid complexes

Rinaldi et al. (2009) suggested that mixed glycolipid complexes may support better binding than pure glycolipids. Neutral glycolipids of the glucosylceramide family are synthesized by sequential addition of sugars to the ceramide core, culminating with the tetrasaccharide form, Gb4 (Table 1). The glucosylceramides display a broad cellular distribution. In contrast, the glycolipid galactosyl ceramide (Gal-Cer), which is synthesized by a different pathway, is found primarily on neuronal tissue [44], [45]. Since selective binding to Stx2 to NAc-Pk is reported, we also evaluated binding of Stx to asialo GM_1_ (aGM_1_) and asialo GM_2_ (aGM_2_) gangliosides [16], [24]. The glycan portion of aGM1 (GalNAcβ1-4Galβ1-4Glc) is similar to NAc-Pk except for the β1–4 instead of α1–4 linkage of the terminal GalNAc residue; aGM_2_ is a derivative of aGM_1_ with an additional Gal residue added with a β1–3 linkage.

**Table 1:** Glycolipids used in this study.

Name (abbreviation, product number)	Structure	Empirical Formula
Glucosyl ceramide (Glc-Cer, 1521)	Glc-Ceramide	C_48_H_93_NO_8_
Lactosyl ceramide (Lac-Cer, 1507)	Galβ1-4Glc-Ceramide	C_53_H_101_NO_13_
Globotriaosyl ceramide, Ceramide trihexoside (Gb3, 1067)	Galα1-4Galβ1-4Glc-Ceramide	C_60_H_113_NO_18_
Globotetraosyl ceramide (Gb4, 1068)	GalNAcβ1-3Galα1-4Galβ1-4Glc-Ceramide	C_68_H_126_N_2_O_23_
Gb3 with non-hydroxy fatty acid side chain (Gb3 –OH, 1513)	Galα1-4Galβ1-4Glc-Ceramide	C_54_H_101_NO_18_
Gb3 with hydroxy fatty acid side chain (Gb3 +OH, 1514)	Galα1-4Galβ1-4Glc-Ceramide	C_54_H_101_NO_19_
Lyso-globotriaosylsphingosine (Lyso-Gb3, 1520)	Galα1-4Galβ1-4Glc-Ceramide	C_36_H_67_NO_17_
Galactosyl ceramide (Gal-Cer, 1050)	Gal-Ceramide	C_48_H_93_N0_8_
Asialo GM_2_ gangliosides (aGM_2_, 1512)	GalNAcβ1-4Galβ1-4Glc-Ceramide	C_56_H_104_N_2_0_18_
Asialo GM_1_ gangliosides (aGM_1_, 1064)	Galβ1-3GalNAcβ1-4Galβ1-4Glc-Ceramide	C_62_H_114_N_2_0_23_

**Figure 4:**
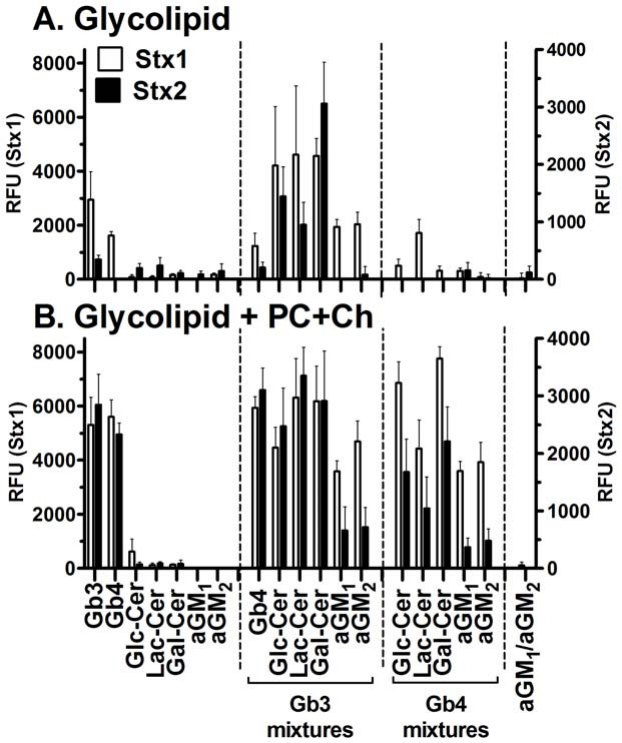
Binding of Stx1 and Stx2 to purified glycolipids and mixtures. Stx binding was assessed by ELISA at 10 nM for both Stx1 (white columns) and Stx2 (black columns) at 4°C. The RFU signal is the mean of three independent experiments and error bars indicate SD. Since different antibodies were used to detect Stx1 and Stx2, two axes are shown. (**A**) **Binding of Stx1 and Stx2 to purified glycolipids and mixtures in absence of Ch and PC**. Mixtures of glycolipids were prepared in methanol at a ratio of 1∶1 and added at 200 ng of total glycolipid per well. (**B**) **Binding of Stx1 and Stx2 to purified glycolipids and mixtures in the presence of Ch and PC**. Mixtures were prepared in methanol at a ratio of glycolipid 1, glycolipid 2, cholesterol, phosphatidylcholine 1∶1∶3∶3 and added at 200 ng of total glycolipid per well.

We examined binding of Stx1 and Stx2 to the neutral glycolipids, alone or in combination (Figure 4A). Stx1 (10 nM) bound to Gb3 and Gb4, but not to Glc-Cer, Lac-Cer, Gal-Cer, aGM_1_ or aGM_2_ (Figure 4A, white bars). Stx1 also bound to 1∶1 mixtures of Gb3 and the other glycolipids, and some mixtures of Gb4. In contrast, strong binding of Stx2 was only observed for Gb3 mixed with Glc-Cer, Lac-Cer or Gal-Cer (Figure 4A, black bars).

In mammalian cells, glycolipids in lipid rafts are arrayed in fluid membranes containing cholesterol (Ch) and phosphatidylcholine (PC). We also examined Stx binding to glycolipid mixtures in the presence of these other membrane components (Figure 4B). Individual glycolipids Glc-Cer, Lac-Cer, Gal-Cer, aGM_1_ and aGM_2_ failed to support binding of either Stx1 or Stx2 even in the presence of Ch and PC (Figure 4B). However, the presence of Ch and PC resulted in increased binding of both Stx1 and Stx2 to Gb3 and Gb4, and both toxins bound to a broader array of glycan mixtures. These initial studies were performed at 4°C. Since membrane fluidity is much greater at physiological temperatures, we repeated these binding studies at 37°C. Incubation at 37°C only resulted in significantly increased binding to Gb3 and Gb4 reflected in higher RFU values (data not shown).

**Figure 5:**
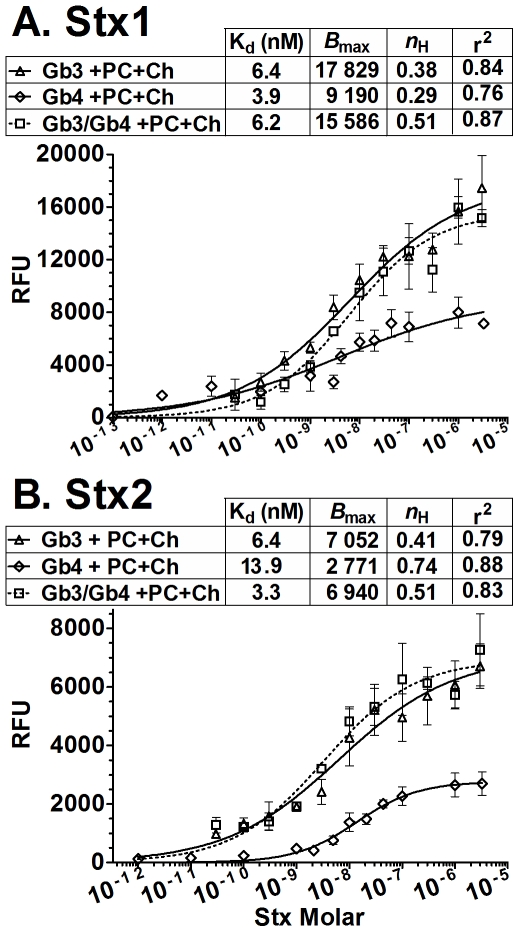
Stx binding to Gb3, Gb4 and Gb3/Gb4 mixture in the presence of cholesterol and phosphatidylcholine. Stx1 (A) and Stx2 (B) toxoid binding was assessed by ELISA at 37°C. As negative controls, toxin was incubated in methanol, PC, Ch, or PC+Ch coated wells. In all experiments, background RFU values obtained in methanol were subtracted from each value. Binding curves were fitted to a one-site specific binding model with Hill coefficients. Symbols represent experimental data, while lines represent the fitted model for that data analyzed with Prism5 (GraphPad software, La Jolla, CA). The RFU signal is the mean of three independent experiments and error bars indicate SD.

The apparent K_d_ of Stx1 and Stx2 for Gb3 and Gb4 in the presence of Ch+PC was assessed at 37°C (Figure 5). The apparent K_d_ of Stx1 for Gb3 in the presence of Ch+PC was 6.4 nM, which is very similar 4.2 nM, the apparent K_d_ of Stx1 for Gb3 at 4°C without Ch+PC (Figure 3). However, the shape of the binding curves was very different, a reflection of the very different hill coefficients (*n*_H_), 1.4 for pure Gb3 at 4°C (Figure 3) versus *n*_H_ = 0.38 for binding to Gb3 with Ch and PC at 37°C (Figure 5A). Since Stx1 has multiple binding sites for Pk, the Hill coefficient of less than 1 seen for Gb3 in the presence of PC and Ch suggests different classes of apparent affinity of the Stx1 toxoid for binding to the plate. This reflects differing levels of avidity rather than differences in individual sites on the Stx1 B-pentamer, likely due to microheterogeneity in the lipid makeup at the plate surface. The avidity of Stx1 for Gb4 was nearly identical to Gb3 alone, with an apparent K_d_ of 3.9 nM compared to 6.4 nM (Figure 5A), but Gb4 supported less binding than Gb3. Binding of Stx1 to Gb3/Gb4 mixture displayed an apparent K_d_ (6.2 nM) very similar to that obtained with either glycolipid alone. Interestingly, in the presence of Ch and PC, Stx2 binding to Gb3, Gb4 or mixtures was very similar to Stx1, both in global affinity (K_d_ of 6.4 nM, 14 nM, and 3.2 nM, respectively) and displaying Hill coefficients of less than 1. The differences in *B*_max_ for Gb4 compared to Gb3 or Gb3/Gb4 for both toxins is significant. This suggests that the number of individual sites on both Stx B-pentamers that can bind Gb4 are presumably lower than the number of sites able to bind Gb3; resulting in a less stringent binding of the B-pentamer to Gb3 compared to Gb4. In support of this hypothesis, similar results (glycans with identical apparent K_d_ but very different *B*_max_ values) were observed for pertussis toxin, an AB_5_ toxin with non-identical B-subunits known to possess structurally and functionally heterogeneous glycan binding sites [41].

### Contribution of the ceramide to Stx binding

**Figure 6:**
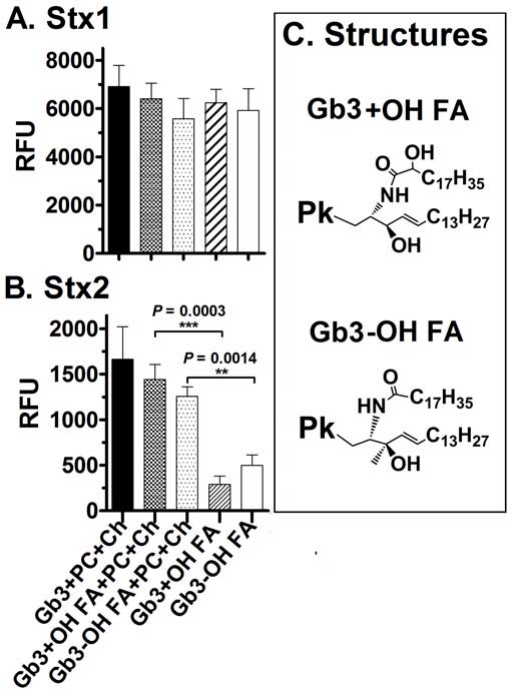
Stx binding to Gb3 analogs. Stx binding was assessed by ELISA at 10 nM for both Stx1 (**A**) and Stx2 (**B**) at 37°C. Gb3 −OH FA, with non-hydroxy Fatty Acid chain; +OH FA with hydroxy Fatty Acid chain. If not specified Gb3 is a standardized mixture that contains both variants with hydroxyl and nonhydroxyl fatty acid chains (Matreya Inc.). As negative controls, toxin was incubated in methanol, PC, Ch, or PC+Ch coated wells. In all experiments, RFU values obtained in methanol were subtracted from each value in order to define a base level. The RFU signal is the mean of three independent experiments and error bars indicate SD.

To determine if the sphingosine residues in the ceramide portion of Gb3 molecule played a role in binding to Stx2, we assessed binding to variants of Gb3 with or without the α-hydroxylated fatty acid (OH FA) in the ceramide (Figure 6). Binding of these variants was compared to the preparation that contains both variants, hydroxyl and nonhydroxyl fatty acid chains, used in Figures 3, 4, 5. Stx1 displayed similar binding to Gb3 regardless of presence of the ceramide hydroxyl or the presence of Ch and PC (Figure 6A). In the absence of PC and Ch, Stx2 failed to bind Gb3 regardless of which form of ceramide hydroxyl was present, and in the presence of PC and Ch bound equally to Gb3 expressing either form of ceramide (Figure 6B). These results demonstrate that the hydroxyl residue in the fatty chain of the sphingosine part of Gb3 does not play a significant role in binding to either Stx1 or Stx2, and agree with previous reports that the OH FA variants of Gb3 display similar binding affinities for both Stx1 and Stx2 [36].

**Figure 7:**
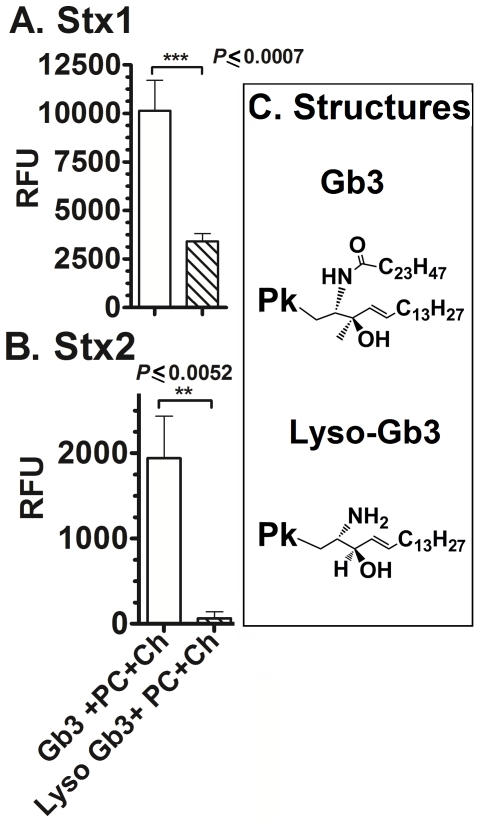
Stx binding to Lyso-Gb3. Stx binding was assessed by ELISA at 10 nM for both Stx1 (**A**) and Stx2 (**B**) at 37°C. As negative controls, toxin was incubated in methanol-coated wells. The RFU signal is the mean of three independent experiments and error bars indicate SD. Statistical differences were calculated by the two-tailed Student's t-test using GraphPad Prism™ 5.

To investigate further the role of the ceramide in Stx binding, we evaluated binding to deacylated Gb3 (Lyso-Gb3). Lyso-Gb3 lacks a carbony group and one fatty acid chain (acyl group) in the sphingosine of Gb3 (Figure 7). Stx1 displayed about a third as much binding to Lyso-Gb3 in the presence of Ch and PC (Figure 7A). Stx2 did not bind to Lyso-Gb3 in the presence or absence of Ch and PC (Figure 7B). These results demonstrate that either the presence of the ketone group or the acyl group in Gb3 is essential for binding to Stx2 at low concentrations of toxin. ELISA probing coated wells with an anti-Gb3 antibody suggest that about 2.8 times more Gb3 than lyso-Gb3 binds to the hydrophobic well in the presence of +PC+Ch; therefore, reduced binding of Stx1 for lyso-Gb3 is likely due to less ligand, and not a reflection of reduced binding affinity of Stx1 to lyso-Gb3 (data not shown). While previous studies reported that Stx1 and Stx2 are able to bind to Lyso-Gb3 by thin layer chromatography [46], receptor binding ELISA [47], [48], [49], or radio-labeled Stx [50], these studies did not compare binding of Lyso-Gb3 to native Gb3. Our results show weak binding of Stx to Lyso Gb3 when compared to native Gb3, which agrees with previous observations [46], [50].

### Contribution of cholesterol to Stx binding

**Figure 8:**
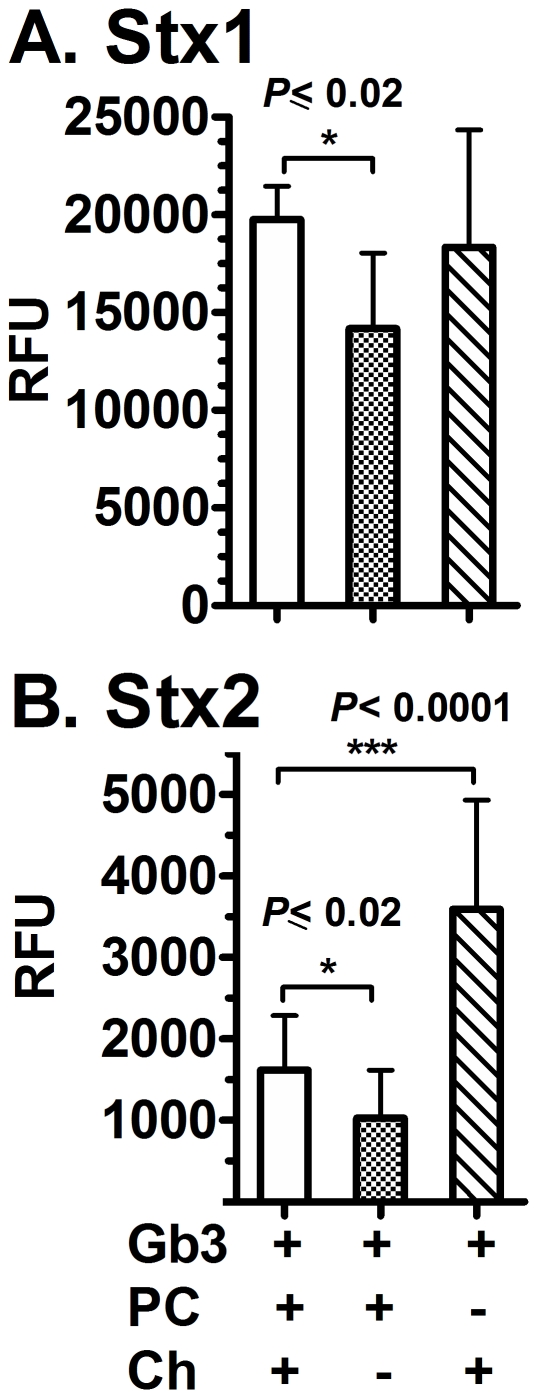
Comparison of Stx binding to Gb3 in absence of cholesterol or phosphatidylcholine. Stx binding was assessed by ELISA at 10 nM for both Stx1 and Stx2 at 37°C as described in Experimental Procedures. As negative controls, toxin was incubated in methanol, PC, Ch or PC+Ch coated wells. In all experiments, RFU values obtained in methanol were subtracted from each value in order to define a base level. The RFU signal is the mean of three independent experiments and error bars indicate SD. Statistical differences were calculated by the two-tailed Student's t-test using GraphPad Prism™ 5.

To determine whether Ch or PC is important to Stx2 binding, we assessed the binding in the absence of either Ch or PC (Figure 8). The absence of cholesterol caused a statistically significant decrease in the binding of Stx1 and Stx2. The presence of cholesterol alone caused a statistically significant increase in the binding of Stx2 to Gb3. These results are consistent with published data by other groups that demonstrate the presence of cholesterol modulates binding to glycosphingolipids [37], [51], [52], [53], [54], and PC does not appear to be required for enhanced binding.

**Figure 9:**
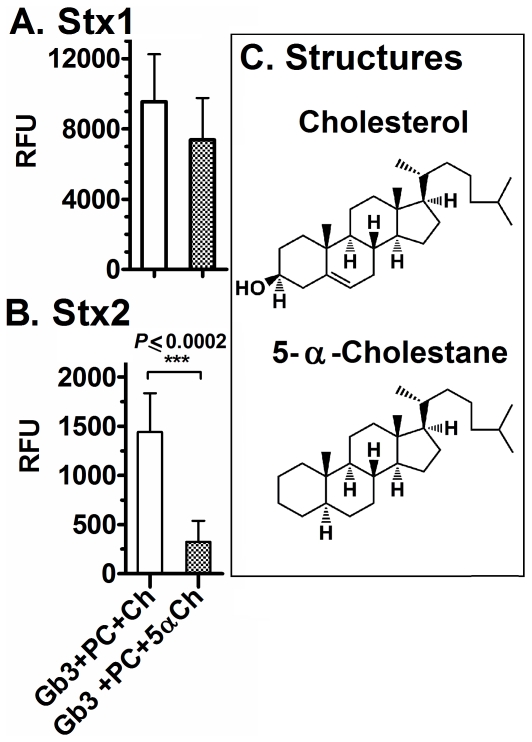
Stx binding to Gb3 in presence of a cholesterol analog. Stx binding was assessed by ELISA at 10 nM for both Stx1 (A) and Stx2 (B) at 37°C. As negative controls, toxin was incubated in methanol-coated wells. The RFU signal is the mean of three independent experiments and error bars indicate SD. Statistical differences were calculated by the two-tailed Student's t-test using GraphPad Prism™ 5.

Yahi et al. reported that cholesterol forms hydrogen bonds with glycosphingolipids by the interaction of the OH of cholesterol (donor group), the NH of sphingosine (acceptor group), and the oxygen atom of the glycosidic bond [acceptor group [52]]. These interactions change the glycolipid conformation and alter glycolipid interactions with proteins. For example, cholesterol has been reported to alter the ability of pathogens such as HIV to interact with the cell [55]. To investigate the role of the OH of cholesterol, we evaluated the binding of Stx1 and Stx2 to Gb3 in the presence of 5-α-Cholestane (5αCh) (Figure 9). This cholesterol analog lacks the OH group at Carbon 3 and has an alkane bond in Carbon 5 (Figure 9C). Stx1 bound equally well in the presence of Ch or 5αCh and PC (Figure 9A). In contrast, 5αCh failed to support binding of Stx2 in the presence of PC (Figure 9B). These results demonstrated that the presence of the OH group in cholesterol plays a role in modulating the binding of Stx2 but not Stx1.

### Stx cellular toxicity in vero protection assay

Little binding of Stx to glycolipids was observed at sub-nanomolar levels (Figures 3 and 5). However, cellular toxicity has been reported to occur at much lower concentrations [12]. Stx causes toxicity by cleaving the 28S rRNA of target cells, thereby inhibiting protein synthesis [8], [9]. We assessed Stx-mediated inhibition of protein synthesis using Vero monkey kidney cells engineered to express a destabilized form of luciferase, Luc2P. Luc2P is targeted to the proteosome for degradation. Since it cannot accumulate in the cell, the amount of luciferase activity is proportional to the current rate of protein synthesis.

To assess the ability of glycolipids to neutralize cellular toxicity, serial dilutions of Stx were incubated in glycolipid-coated microtiter plates at 37°C for 1 hour, essentially as described in Figure 5. The supernatant containing unbound toxin was transferred to plates containing the Luc2P Vero cells. Protein synthesis inhibition was assessed after 4 hours of incubation with the toxin.

**Figure 10:**
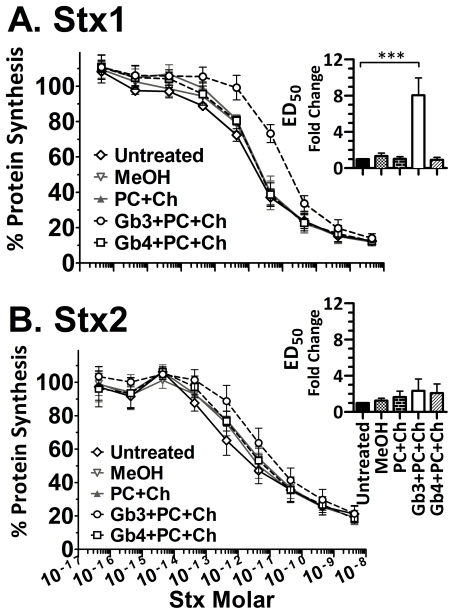
Vero protection studies. Stx cellular toxicity was assessed using luciferase activity of Luc2p Vero cells treated with dilutions of Stx1 (A) or Stx2 (B) pre-incubated with glycolipid mixtures as described in Figure 5. As negative controls, toxin was untreated or incubated in methanol-coated wells or PC+Ch. The results are the average of three independent experiments. Statistical difference was calculated between untreated control and Gb3+PC+Ch treatment by the two-tailed Student's *t*-test using GraphPad Prism™ 5 (***, *P* = 0.0002).

In this assay, the ED_50_ for untreated Stx1 was 0.3×10^−11^, and the ED_50_ for untreated Stx2 was 5×10^−11^. Pre-incubation of Stx1 in wells treated with methanol (Figure 10A, open inverted triangles) or PC+Ch (Figure 10A, open triangles) did not result in decreased toxicity, as seen by no change in ED_50_ compared to the untreated control (Figure 10A, insert). Pre-incubation with Gb4+PC+Ch (Figure 10A, open squares) was not able to protect Vero cells. However, pre-incubation of Stx1 with Gb3+PC+Ch (Figure 10A, open circles) resulted in significantly reduced toxicity, with about a 10-fold increase in the ED_50_ compared to untreated Stx1 (Figure 10A, insert). In contrast, Stx2 was not neutralized by any of the treatments since there were no significant differences in the ED_50_ values for treated or untreated toxin (Figure 10B: insert).

## Discussion

The present study provides insights into the difference in receptor recognition by Stx1 and Stx2. While Stx1 binds with similar affinity to the Pk glycan and the Gb3 glycolipid (Figure 5), Stx2 does not recognize Pk alone, but can bind in the context of Gb3 glycolipid and other molecules (Figures 4, 5). We found that Stx1 can also bind to the P tetrasaccharide (Figure 1) and the Gb4 glycolipid (Figure 4, 5), which has not been reported previously. While Stx2 did not bind P glycan, it could bind to the glycolipid Gb4 (Figure 5). Given the differences in the ability of Stx1 and Stx2 to recognize glycan, it is intriguing that both toxins bind to the glycolipids Gb3 and Gb4 with nearly identical affinity when PC and Ch are present (Figure 5).

Unlike Stx1, Stx2 binding to Gb3 is critically dependent on the presence of other compounds, either another glycolipid such as Gal-Cer or Ch (Figure 4). It is interesting to note that Stx2 but not Stx1 is associated with neurologic damage, and Gal-Cer is highly expressed on neuronal tissues [3], [56]. The second component could enhance binding of Stx2 to Gb3 either by directly contacting the toxin or by inducing Gb3 to assume a conformation more favorable for Stx2 binding. Ch has been shown to form hydrogen bonds with the ceramide on glycolipids, leading to conformational changes that make cells more susceptible to infection with HIV [51], [52],[53]. We do not know if this mechanism is responsible for increased binding of Stx2 in the presence of Ch. However, we do not believe that the greatly improved binding of Stx2 to 1∶1 mixture of Gb3 and Gal-Cer in the absence of Ch is achieved through conformational changes, since the ceramide of Gal-Cer and Gb3 is identical. An explanation that would account for the increased binding of Stx2 in the presence of Ch and Gal-Cer is that these molecules provide additional binding contacts. Stx2 could form hydrogen bonds with the galactose on Gal-Cer or with cholesterol.

In addition to the potential for additional binding contacts, the lower Hill coefficients observed for both Stx1 and Stx2 in the presence of cholesterol suggest that different classes of avidity are displayed on the plate surface, presumably due to heterogeneity in the distribution of the molecules. Inclusion of PC and Ch may favor formation of lipid microdomains that support Stx binding to differing degrees, resulting in broadened binding curves due to overlapping ranges of avidity depending on the localized geometry of the glycans and how they interact with the binding sites on the toxin. Clusters of glycolipids whose geometry precisely matches the binding sites within the toxin would allow maximum apparent affinity, and the reduced fluidity of the membrane upon addition of Ch would increase the lifetime of such localized glycolipid populations. This phenomenon may have important implications for the in vivo biological activity of the toxin, since such broadened binding curves exhibit detectable binding at very low toxin concentrations.

Studies with chimeric toxins where the Stx1 and Stx2 A- and B-subunits were reassorted demonstrated that potency tracks with the B-subunit of Stx2 [15], [17], [26], strongly suggesting that potency is determined by which cells are targeted, which is determined by receptor usage. However, the current results do not explain the difference in potency of Stx1 and Stx2. An enormous disparity exists between the binding observed using biochemical assays compared to cellular susceptibility. The K_d_ values of Stx1 and Stx2 to Gb3 from this and previously published studies [15], [23] generally range between 10^−7^ M and 10^−9^ M. The concentration of toxin in blood at 50% lethal dose in mice is approximately 10^−9^ M for Stx1 and 10^−11^ M for Stx2 [12]. However, both Stx1 and Stx2 are toxic to primary human renal proximal tubular epithelial cells of the kidney with an ED_50_ of about 10^−13^ M [12] and to the Vero monkey kidney cell line with an ED_50_ of about 10^−11^ M (Figure 10). Since we are unable to observe any binding in vitro at these low doses, we examined the ability of toxin preincubated with glycolipid to protect Vero cells from Stx-mediated inhibition of protein synthesis. Even though nearly identical K_d_s were observed for Stx1 and Stx2 binding to Gb3 and Gb4 (Figure 4), Stx1 but not Stx2 was neutralized by preincubation with Gb3 mixed with PC+CH (Figure 10). These studies suggest that the in vitro glycolipid system replicates most of the elements need for cellular binding of Stx1, but not Stx2.

Several properties of living cells could allow for toxin activity at concentrations where no binding occurs in biochemical systems. One major difference is the membranes of living cells are highly fluid and can form invaginations or protrusions, which cannot be formed by membrane components bound to the rigid surface of microtiter plates. Stx1 has been shown to induce tubular membrane invaginations both in living cells and model membranes [61], and the high concentration of Stx1 in the tubules could drive toxin binding. Currently, there are no reports that Stx2 can induce tubular invaginations.

In addition to membrane plasticity, living cells may express other molecules which bind Stx1 or Stx2 with a higher affinity than Gb3. In the glycan array (Figure 2), Stx1 bound better to glycans containing GlcNAc at the third position instead of Glc (Galα1-4Galβ1-4**GlcNAc** versus Galα1-4Galβ1-4**Glc**). In other published reports [16], [24], Stx2 preferred a Pk mimic (NAc-Pk: NAcGalα1-4Galβ1-4Glc) to native Pk. While these preferred glycans are not found on glycolipids, both are found on glycoproteins, and accumulating reports suggest that Stx may engage protein receptors. In 1999, Katagiri et al. were the first to report that Stx induced activation of tyrosine kinase within minutes of binding to a cell [57]. Recently, treatment with the B-pentamer from either Stx1 or Stx2 was shown to promote release of von Willebrand factor (VWF) from endothelial cells [58] by a process that is dependent on Gb3 and cholesterol, and requires caveolin-1, but not clathrin, and Stx2B can initiate activation of the coagulation cascade in animal models of disease [59]. Furthermore, it has recently been shown that Stx1B and Stx2B use different signaling pathways to promote VWF release [58] Activation of VWF release by Stx1B is associated with transient elevation of intracellular calcium, and requires both phospholipase C and protein kinase C. In contrast, activation of VWF release by Stx2B requires protein kinase A, which is activated in a cAMP-independent manner. Stx could activate a signaling pathway by binding to a protein receptor in a manner which mimics agonist activation. Alternatively, Stx could promote receptor activation by a lectin-like mechanism. Lectins activate signaling pathways that respond to receptor-clustering. Like Stx, lectins possess multiple glycan-binding sites, and can crosslink receptors via N- or O-linked glycans present on receptor proteins. The presence of protein receptors could enhance Stx bind to cells. However, it is important to recognize that living cells can internalize the toxin, and internalized toxin in a cellular system is equivalent to irreversible binding in a biochemical system.

Important questions regarding the pathogenesis of Stx-mediated disease remain unanswered. Why is Stx2 more likely to cause fatal disease than Stx1? Why are children more susceptible than adults? Is Stx-mediated killing of kidney epithelial cells more important than Stx-mediated activation of the clotting cascade by kidney endothelial cells? Since hemolytic uremic syndrome patients who also display neurologic symptoms are more likely to succumb to fatal disease [56], [60], does Stx target the nervous system? Currently, only supportive care is available for patients with Stx-mediated disease. A detailed understanding of toxin binding preferences would allow us to identify the cells, organ systems, and even individuals that are most susceptible to the toxin. Such understanding is essential for the development of effective treatment strategies.

## Materials and Methods

### Production of recombinant Stx toxoids and B-pentamers

Toxin-encoding genes were PCR amplified and cloned into the expression plasmids, as outlined in Table 2 and 3. The sequence of all inserts was verified. To generate the Stx2 toxoid expression construct (pTSG218), the inactivated *stx2* operon from pNR100 [40] was cloned as single PCR product. To generate the Stx1 toxoid expression construct, pTSG214 containing the *stx1A* and *B* genes in tandem, tyrosine 77 and glutamic acid 167 of *stx1A* were sequentially replaced with serine and glutamine, respectively using the QuickChangeTM protocol (Stratagene) generating pTSG213. *stx1B* was excised from pTSG211 with *Xba*I *and Not*I and cloned into the *Not*I and *Spe*I site of pTSG213.

**Table 2:** Plasmids used in this study.

Plasmid	Genotype	Vector/PCR Template (Reference)
pTSG210	Stx1A-WT	pETSecS3 [41]/pMFUC-17 [26]
pTSG211	Stx1B-WT	pETSecS3/pSW09 [40]
pTSG212	Stx1A-Y77S	pTSG210 (This study)
pTSG213	Stx1A-Y77SE167Q	pTSG212 (This study)
pTSG214	Stx1A-Y77SE167Q+Stx1B-WT	pTSG213 (This study)
pTSG218	Stx2A-Y77SE167Q+Stx2B	pETSecS3/pNR100 [40]
pTSG230	Stx2B-WT	pETSecS3 [41]/pMFCU-21 [26]

**Table 3:** Primers used in this study.

Cloning Primers	
Name	Sequence
5′ Stx1A *Nde*I	AACATATGATGAAAATAATTATTTTTAGAGTGC
3′ Stx1A *Spe*I	ATACTAGTTCAACTGCTAATAGTTCTGCGC
5′ Stx1B *Nde*I	AACATATGATGAAAAAAACATTATTAATAGCTGC
3′ Stx1B SpeI	ATACTAGTTCAACGAAAAATAACTTCGCTG
5′ Stx2A *Nde*I	AACATATGATGAAGTGTATATTATTTAAATGGG
3′Stx2A *Spe*I	ATACTAGTTCAGTCATTATTAAACTGCACTTC
5′Stx2B *Nde*I	GGAATTCCATATGAAGAAGATGTTTATGGCGG
3′Stx2B *Spe*I	GGACTAGTTCAGTCATTATTAAACTGCACTTCAG

Site-Directed Mutagenesis Primers	
Name	Sequence
5′ Stx1A-Y77S	GGTTTAATAATCTACGGCTTATTGTTGAACGAAATAATTTAAGTGTGACAGGATTTGTTAACAG
3′ Stx1A-Y77S	CTGTTAACAAATCCTGTCACACTTAAATTATTTCGTTCAACAATAAGCCGTAGATTATTAAACC
5′ Stx1A-E189Q	CGGTTTGTTACTGTGACAGCTCAGGCTTTACGTTTTCGGC
3′Stx1A-E189Q	GCCGAAAACGTAAAGCCTGAGCTGTCACAGTAACAAACCG

Proteins were expressed from cold-induced cultures as previously described [16], [61], with the following modifications. Briefly, logarithmic phase cultures were cooled to 8°C; expression of recombinant toxoid and protein folding genes was induced by addition of IPTG (0.1 mM) and ethanol (2%), respectively. After overnight incubation with shaking at 20°C, the cells were harvested by centrifugation, and lysed by gentle shaking with 4 M urea for 30 minutes. Cellular debris was removed by centrifugation. The extract was dialyzed, and concentrated as a 40–70% ammonium sulfate fraction. Toxoids were further purified using combinations of AffiGel Blue affinity chromatography (Bio-Rad, CA), ion exchange, or size exclusion chromatography. Pigeon egg white affinity chromatography [61] was used for Stx1 toxoid. Protein was quantified using bicinchoninic acid protein assay (Pierce, IL). Purity of toxoid was verified by the presence of only two bands corresponding to the A-and B-subunits on Coomassie stained 8–16% polyacrylamide gels (Lonza) loaded with 1 µg of protein.

### Isothermal Titration Calorimetry (ITC)

ITC experiments were performed in a Microcal VP-ITC microcalorimeter at 25°C in buffer containing 20 mM HEPES at pH 7.4 and 150 mM NaCl. Stx1B and Stx2B were dialyzed into this buffer, and powdered Pk trisaccharide and P tetrasaccharide glycans were resuspended in dialysate to achieve a buffer match. All experiments were performed with Stx B-subunits in the microcalorimeter cell at 238–300 µM concentration, and glycans in the syringe at 50 mM concentration. The titrations consisted of a total of forty 7-µl injections, spaced 120 seconds apart. Protein concentrations were determined based on the UV absorbance at 280 nm and molar extinction coefficients of the Stx1B and Stx2B monomers (8,605 M^−1^cm^−1^ and 14,105 M^−1^cm^−1^, respectively). Data were analyzed in ORIGIN using a one-site binding model with fixed n = 1 per B subunit (the fixed parameter was required to achieve convergence of the fit). The K_d_ values reported are the average of two replicates.

### Glycan array studies

Stx1 (2.84 µM) and Stx2 (0.64 µM) toxoids (obtained from the Biodefense and Emerging Infectious Diseases Research Resources Repository, Manassas, VA) were submitted to the Consortium for Functional Glycomics (CFG) to assess glycan binding specificity. The Mammalian Printed Array Version 4.1 holds 465 different glycans consisting of natural and synthetic mammalian glycans. Toxin binding was detected using rabbit polyclonal antibody to Stx (Meridian Bioscience, Cincinnati, OH) and fluorescently labeled anti-rabbit IgG Alexa 488 antibody which was supplied by the CFG. The array consists of six replicates of each glycan, and relative binding was expressed as mean relative fluorescence units (RFU) of four of the six replicates after removal of the highest and lowest values. Binding data can be accessed at the CFG website (http://www.functionalglycomics.org/).

### Glycolipid ELISA

Glycolipids and lipids (Table 1) were purchased from Matreya Inc. (Pleasant Gap, PA). Pure glycolipids were suspended in chloroform and diluted in methanol as previously described [31]. Mixtures of glycolipids were prepared in a molar ratio of 1∶1. Mixtures of glycolipids with cholesterol (Ch) and phosphatidylcholine (PC) were prepared in a molar ratio of 1∶3∶3 as previously described [15]. Single or mixed glycolipids with or without Ch and PC were added to wells of hydrophobic Microtiter® plates (Microfluor® 1, Thermo scientific) and dried for 30 hours in a fume hood. As negative controls, methanol alone, PC, Ch or PC+Ch were added to wells. In all experiments, background RFU values obtained in methanol were subtracted from each value. Except were indicated, all steps were performed at 4°C. Prior to use, the plates were blocked for 1 hour with phosphate buffered saline (PBS; 8.1 mM Na_2_HPO_4_, 1.5 mM KH_2_PO_4_, 128 mM NaCl, 2.7 mM KCl), pH 7.4, containing 2% (w/v) bovine serum albumin (BSA). Dilutions of Stx toxoid were added and incubated for 1 hour, followed by sequential incubation with primary antibody against Stx1 or Stx2 (rabbit polyclonal serum, Meridian Bioscience, Cincinnati, OH) and peroxidase-conjugated goat anti-rabbit IgG (MP Biomedicals, Solon, OH). Wash steps were carried out using cold PBS pH 7.4 containing 1% (w/v) BSA. Finally, plates were developed with QuantaBlu® fluorogenic peroxidase substrate (Pierce, Rockford, IL) and read. Binding curves and analysis were performed using Prism 5.0 (GraphPad Software, La Jolla, CA).

### Vero protection studies

Microtiter plates were coated with glycolipid as described above. Wells treated with methanol alone, PC+Ch alone, or not pre-treated served as negative controls. Unbound surfaces on the wells were blocked with Minimal Essential Medium 1× (*Invitrogen*™) supplemented with 10% fetal bovine serum, vitamins (*Sigma-Aldrich*™) and glutamine (*Sigma*™), and washed with PBS. Stx1 and Stx2 (*Biodefense and Emerging Infectious Diseases Research Resources Repository, Manassas, VA*) were serially diluted in PBS and added to the wells, starting with 10^−8^ M of toxin. The toxin was incubated at 37°C for 1 hour. After incubation, the toxin was removed from the wells and added to tissue culture treated 96 well plates (*Corning Inc.™*). The amount of residual toxin was determined as previously described [12], [62] by measuring protein synthesis inhibition using Luc2P Vero cells engineered to express destabilized luciferase [62] . Briefly, Luc2P Vero cells were added at 10^4^ cells per well. After 4 hours of incubation at 37°C and 5% CO_2_, the cells were washed with PBS and 25 µl/well of *SuperLight* luciferase substrate was added and luminescence was measured. The results were reported as percentage of maximum signal from PBS control cells incubated without any toxin. The effective dose to inhibit 50% of protein synthesis (ED_50_) was calculated using the two points above and below the midpoint and normalized against the untreated control.
